# Supplementary material for: Cloud BioLinux: pre-configured and on-demand bioinformatics computing for the genomics community
Source: BMC Bioinformatics. 2012 Mar 19;13:42. doi: 10.1186/1471-2105-13-42 (PMC3372431; doi:10.1186/1471-2105-13-42)
Supplement: Additional file 1 — Supplementary 1 Cloud BioLinux software documentation in the form of a mini, self-contained website. Users need to download and uncompress the .zip file, and open through a web browser the "index.html" file available on the main directory. (ZIP 1823 kb). [file 1471-2105-13-42-S1.ZIP › Cloud-BioLinux-Package-Documentation/docs/mira.html]

Bio-Linux Software Documentation Pages

Back to search form

## mira

|  |  |
| --- | --- |
| Name | mira |
| Description | **MIRA** is described as a swiss army knife for sequence assembly.  Mira performs true hybrid de-novo assemblies using reads gathered through Sanger, 454 or Illumina sequencing technologies. It also contains integrated editors for Sanger and 454 sequences.  Mira is well regarded, comprehensive sequence assembly software. To learn more about mira, please refer to the remote documentation. |
| Homepage | http://chevreux.org/projects\_mira.html |
| Remote Documentation | http://chevreux.org/mira\_examples.html      http://chevreux.org/mira\_features.html      http://chevreux.org/mira\_manuals.html |
